# Assessing countermeasures during a hepatitis A virus outbreak among men who have sex with men

**DOI:** 10.1186/s12976-021-00150-1

**Published:** 2021-10-11

**Authors:** Ryohei Saito, Akifumi Imamura, Hiroshi Nishiura

**Affiliations:** 1 Graduate School of Medicine, Hokkaido University, Kita 15 Jo Nishi 7 Chome, Kita-ku, Sapporo-shi, Hokkaido 060-8638 Japan; 2 Department of Infectious Diseases, Tokyo Metropolitan Cancer and Infectious Diseases Center Komagome Hospital, 3-18-22 Honkomagome, Bunkyo-ku, Tokyo, 113-8677 Japan; 3 Kyoto University School of Public Health, Yoshidakonoecho, Sakyo-ku, Kyoto-shi, Kyoto, 606-8503 Japan

**Keywords:** Intervention, Awareness, Epidemic, Mathematical model, Sexually transmitted infection

## Abstract

**Background:**

A hepatitis A epidemic occurred among men who have sex with men (MSM) in Japan in 2017–2018. In this study, we employ a parsimonious mathematical model to epidemiologically investigate the dynamics of infection, aiming to evaluate the effectiveness of campaign-based interventions among MSM to raise awareness of the situation.

**Methods:**

A mathematical model describing a mixture of human-to-human transmission and environmental transmission was fitted to surveillance data. Taking seasonally varying environmental transmission into account, we estimated the reproduction number of hepatitis A virus during the course of epidemic, and, especially, the abrupt decline in this reproduction number following campaign-based interventions.

**Results:**

The reproduction number prior to the countermeasures ranged from 2.6 to 3.1 and then began to decrease following campaign-based interventions. After the first countermeasure, the reproduction number decreased, but the epidemic remained supercritical (i.e., *R*_*t*_ > 1). The value of *R*_*t*_ dropped well below one following the second countermeasure, which used web articles to widely disseminate information about the epidemic risk.

**Conclusions:**

Although the effective reproduction number, *R*_*t*_, changes because of both intrinsic and extrinsic factors, the timing of the examined countermeasures against hepatitis A in the MSM population was consistent with the abrupt declines observed in *R*_*t*_. Even without vaccination, the epidemic was brought under control, and risky behaviors may have been changed by the increase in situation awareness reached through web articles.

**Supplementary Information:**

The online version contains supplementary material available at 10.1186/s12976-021-00150-1.

## Background

Hepatitis A, an acute viral infectious disease, is a type of hepatitis that frequently involves long-lasting fever, nausea, vomiting, diarrhea, abdominal pain, and strong fatigue, with or without jaundice [1], although the infection is sometimes asymptomatic. The disease tends to be more serious in older people than in younger people, and most children aged under 6 years are asymptomatic [2]. The disease is caused by hepatitis A virus (HAV), which is transmitted via the fecal–oral route and also by eating contaminated food [2]. In particular, poorly or insufficiently cooked shellfish often acts as the source of infection [3]. HAV is widely prevalent in the environment, and it is able to survive a variety of food-production procedures [4]. The transmission involves seasonal variation, with the majority of infections occurring in the spring and summer [5]. Although hepatitis A has become rarer in industrialized countries, it still spreads sporadically, and the virus is globally prevalent, with periodic outbreaks. The ecological dynamics remain largely unknown but are believed to be regulated by the concentration and habitat of shellfish [6]. In Asia, a large outbreak of HAV occurred in Shanghai in 1988, involving 300,000 infections [7]. People who recover from natural infection with HAV develop life-long immunity [8], and a safe and effective (inactivated or live-attenuated) vaccine is available [9]. The main public health efforts to control the infection are supplying safe water and food, improving hygiene, encouraging handwashing, and immunizing those who are susceptible [5]. In general, the incubation period ranges from 14 to 28 days [10]. Serological diagnosis is made by confirming elevated serum immunoglobulin M antibodies [2]. There is no specific treatment for hepatitis A, and only supportive care is routinely offered [2, 4].

**Fig. 1:**
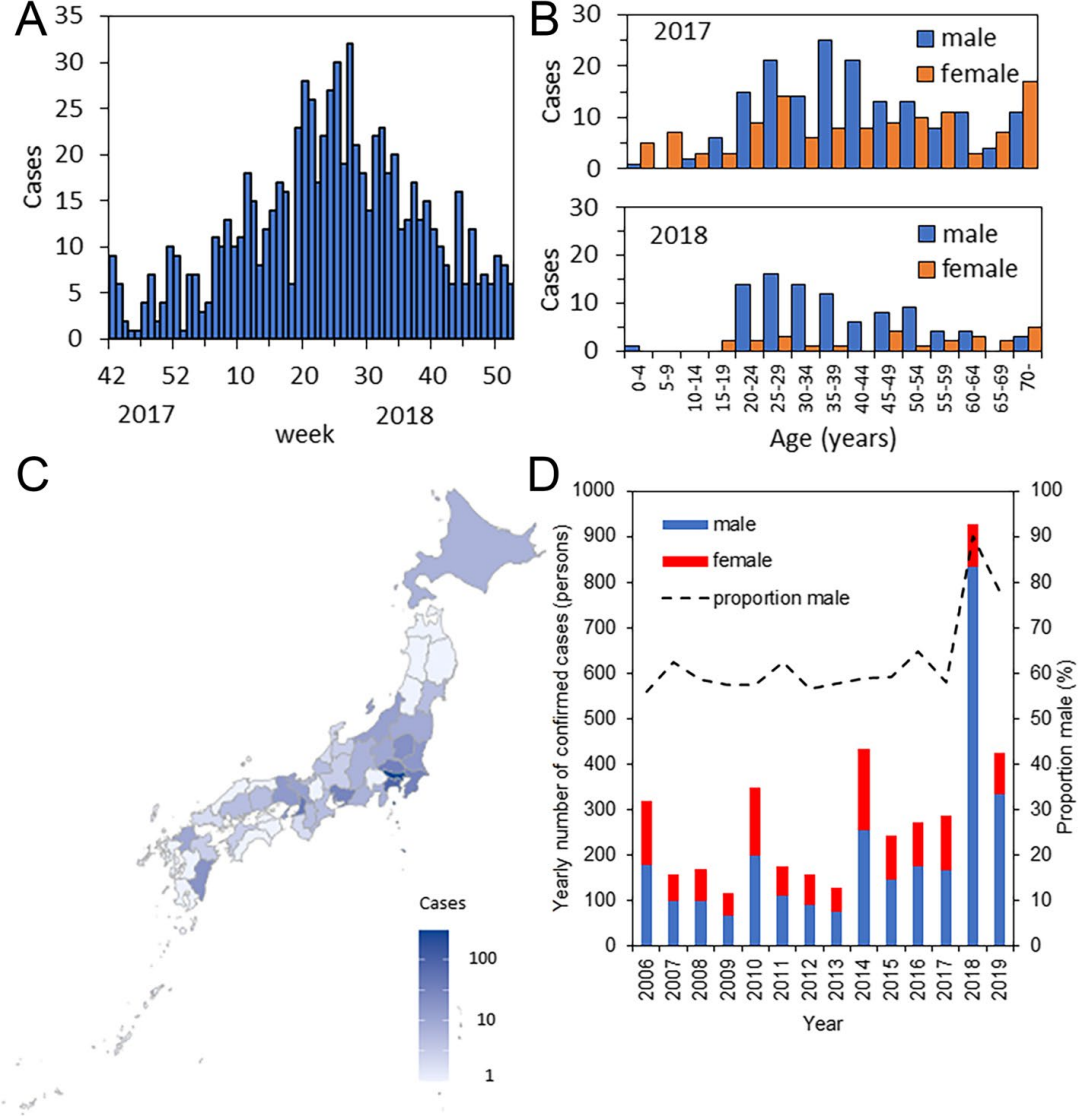
Epidemic curve of hepatitis A in Japan in 2018 by week, age, and sex distribution. **A** Temporal distribution of the incidence (serologically diagnosed cases) from 2017 to 2018. **B** Age distribution of serologically diagnosed cases. The data from 2017 are based on whole-year notifications, but the 2018 data were gathered only up to July 26th. In 2017, the age distribution was fairly flat for women and peaked at 35–39 years for men, and the male-to-female ratio was not particularly high. In contrast, most cases in 2018 occurred in young men aged 20–55 years. **C** Spatial distribution of serologically diagnosed cases from 2017 to 2018. Many cases were diagnosed in the Kanto region, which includes the Tokyo metropolitan area, followed by Osaka Prefecture, the third largest prefecture in western Japan. **D** Sex distribution of confirmed cases from 2006-19. Proportion male is measured on right vertical axis

Infection with HAV can occur by direct mucous-to-mucous contact, especially among men who have sex with men (MSM) [2]. MSM sex tourism across national borders is recognized as an important avenue of introducing new outbreaks [11], and many outbreaks of hepatitis A occur among MSM. The timing of hepatitis A outbreaks has involved a certain time lag as they have moved across the globe, with a surge of patients occurring among MSM from 2015 to 2017 in Taiwan [12–14], from 2016 to 2017 in Europe [15–29], from 2016 to 2018 in the United States [30, 31], and in 2018 in Japan [11, 32]. Spatial spread (e.g., from Taiwan and Europe to Japan) has been partly demonstrated via genome sequencing [11, 32]. In 2018, Japan experienced a large outbreak in which the majority of cases were young men (Fig. 1A and B). Recent sporadic cases have occurred among MSM in previous decades [33], and it was not difficult to anticipate another outbreak in 2018. A particularly substantial increase in disease incidence was seen in Tokyo (Fig. 1C) and Osaka [32]. Fortunately, the outbreak was brought under control in about 6 months (Fig. 1A). The confirmed cases in Japan from 2006-19 were dominated by men, indicating that the transmission was highly heterogeneous, fueled by the population of MSM, and women was perhaps dead-end host or acquired infection from environment (Fig. 1D).

Hepatitis A is a notifiable disease; thus, Japan collects data on counts of all diagnosed cases [34], and we were able to use the publicly available data to assess interventions from 2017 to 2018. In this study, we employed a parsimonious mathematical model to epidemiologically investigate the dynamics of infection. The purpose of the study was to evaluate the effectiveness of interventions by quantitatively assessing the impact of campaigns that were implemented among MSM in terms of reducing the incidence of hepatitis A infection. We also examined the transmission dynamics in Japan, referring to published evidence on the transmission dynamics of HAV in the past [35–43].

## Methods

### Epidemiological data

In the Japanese Law of Infection Control, hepatitis A is a class 4 notifiable disease and must be reported within 24 hours of confirmatory diagnosis. Confirmatory diagnosis is made by polymerase chain reaction or serologically by elevated immunoglobulin M titer. After anonymizing the data, the National Institute of Infectious Diseases reports a weekly record of the frequency of cases [34]. During the course of the outbreak of HAV from 2017 to 2018, various preventive campaigns were conducted among MSM. In particular, special efforts were made from February 13 to May 5, 2018 (see Table 1 for a detailed chronology). For instance, special pamphlets were widely distributed on February 29, and an online article warning readers about the outbreak that was published on March 30 was accessed over 120,000 times. Because these campaigns were the most likely interventions to have increased recognition of the outbreak, we labeled February 29 as *t*_1_ and March 30 as *t*_2_ for use in our modeling analysis. That is, the most strengthened effort to let people recognize the outbreak was made with 1000 pamphlets on *t*_1_ (February 29), and the costly approach via web article was conducted on *t*_2_ (March 30).

**Table 1:** Chronology of interventions against the hepatitis A virus outbreak among MSM in Japan, 2018

Calender time	Time parameter	Events
13-Feb-18		Submission of data of pamphlet
		Published a web article
		Information via twitter
26-Feb-18		Distributed 305 posters
29-Feb-18	t_1_^b^	Printed and distributed 1000 pamphlets
9-Mar-18		Distributed 165 posters
9-Mar-18		Published a web article in HIV map (which is accessed by substantial number of men who have sex with men)
10-Mar-18		Distributed 20 posters
12-Mar-18		Distributed 10 posters
17-Mar-18		Put an article on a magazine
30-Mar-18	t_2_^c^	Published a web article
3-May-18		Distributed 20 pamphlets
5-May-18		Distributed (i) 165 and (ii) 1815 pamphlets for (i) delivery health project, Shinjuku 2-chome and bar and (ii) Tokyo Rainbow Pride Parade (for LGBTQ) in Yoyogi Park, respectively

*MSM* men who have sex with men

t_1_ was the time at which the first campaign was implemented. t_2_ was the time at which the second campaign was implemented

### Mathematical model

**Fig. 2:**
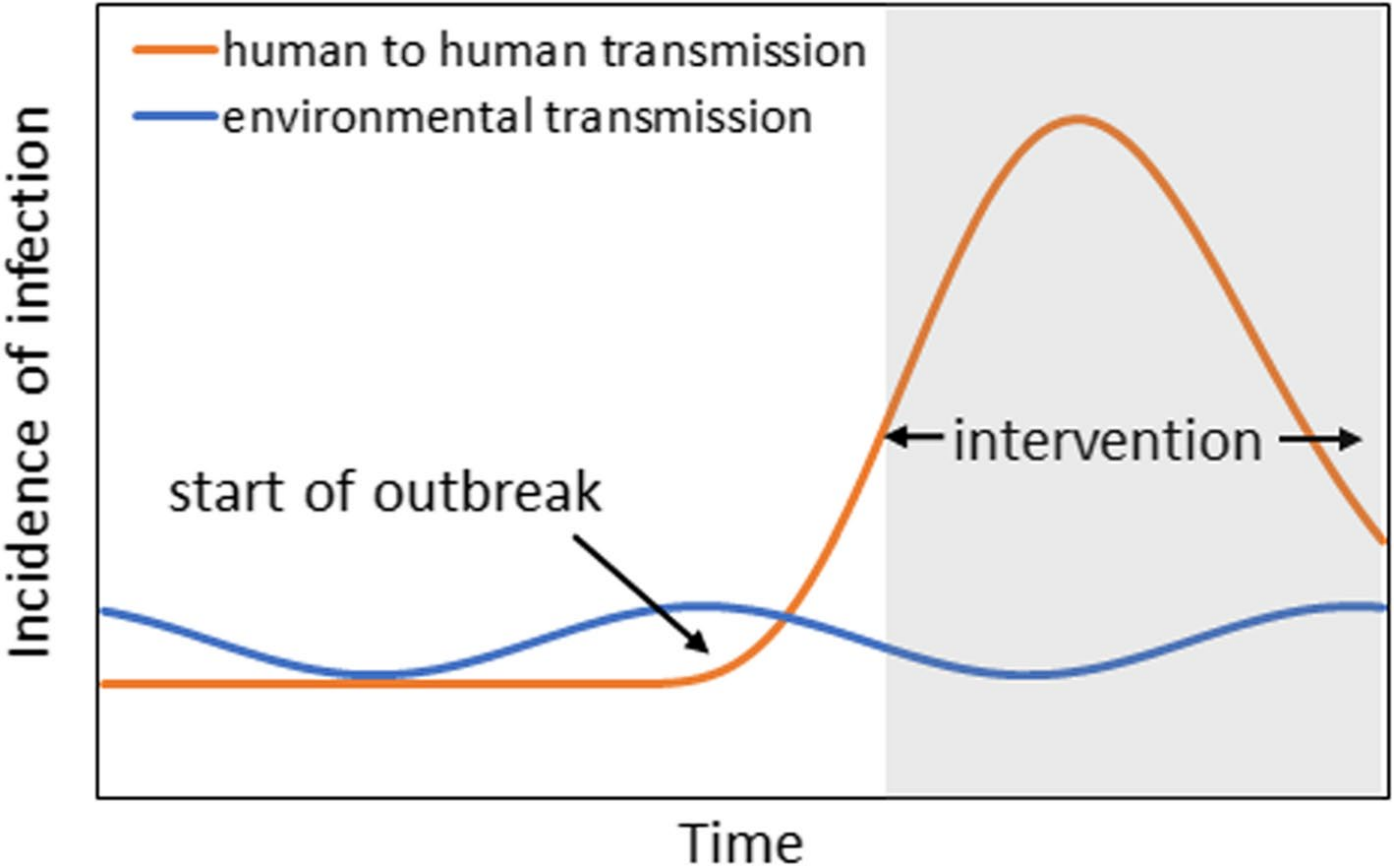
Interpreting the observed patterns of the epidemic curve. We assumed that the observed epidemic curve could be decomposed into environmental transmission and human-to-human transmission. Environmental transmission, which varies seasonally, corresponds to a sine-like curve. The other curve represents human-to-human transmissions among men who have sex with men

HAV is transmitted via environmental and human-to-human routes. To account for this fact, we assumed that the observed epidemic curve can be decomposed into two distinct types of transmission (i.e., those infected via the environmental route and those infected via human-to-human transmission; Fig. 2). We thus described the mixture of these two mechanisms and fitted our model to the data. Environmental transmission is governed by a hazard that varies seasonally, with a summer peak. The other type, human-to-human transmission, is caused by sexual contact among men, and a renewal equation was used to describe the dynamics. Modeling the incidence of infection, the epidemic curve, described using the date of illness onset, was calculated as the convolution of the incidence and the incubation period.

Seasonal variation in the environmental transmission was modeled using the trigonometric function for periodic changes, which we assumed to be a good approximation. This model is described as follows:

1 $$a(t)={a}_0+{\sum}_{k=1}^n{a}_k{\sin}\left(\frac{2\pi kt}{52}+{b}_k\right),$$

where *a*(*t*) is the number of newly infected cases caused by environmental transmission at calendar time *t* (*weeks*), *n* is the number of terms in the trigonometric function, and *a*_0_, *a*_*k*_, and *b*_*k*_ are the estimated parameters.

Let *j*(*t*) be the number of cases newly infected via human-to-human transmission at calendar time *t*. They are produced by people infected in the past, and, on average, each primary case generated *R*_1_ secondary cases:

2 $$j(t)={R}_1{\int}_0^tj\left(t-s\right)g(s) ds.$$

Here, *g*(*s*)is the discretized probability density function of the generation time, derived as *g*(*s*) = *G*(*s*) − *G*(*s* − 1), where *G*(*s*) is the cumulative distribution function of the generation time, assumed to be a lognormal distribution with a median of 27.5 days and a standard deviation of 4 days [10]. When the first countermeasure was implemented at time *t*_1_, the reproduction number changed from *R*_1_ to *R*_1_*ε*_1_(0 < *ε*_1_ < 1). Therefore, the renewal equation also changed to

3 $$j(t)={R}_1{\varepsilon}_1{\int}_0^tj\left(t-s\right)g(s) ds.$$

Similarly, when the second countermeasure was conducted at time *t*_2_, the reproduction number changed from *R*_1_*ε*_1_ to *R*_1_*ε*_1_*ε*_2_(0 < *ε*_2_ < 1). Therefore, the renewal equation changed to

4 $$j(t)={R}_1{\varepsilon}_1{\varepsilon}_2{\int}_0^tj\left(t-s\right)g(s) ds.$$

Alternatively, we also examined the case in which intervention effects were time-dependent. That is, we considered ${R}_1{\varepsilon}_1^{t-{t}_1}$ rather than *R*_1_*ε*_1_ and also ${R}_1{\varepsilon}_1^{t_2-{t}_1}{\varepsilon}_2^{t-{t}_2}$ rather than *R*_1_*ε*_1_*ε*_2_.

Taking the sum of environmentally and human-to-human transmitted cases—*a*(*t* − *s*) and *j*(*t* − *s*), respectively—and convoluting it with the incubation period, we obtained the expected number of cases with illness onset at calendar time *t*, *E*[*c*_*t*_]:

5 $$E\left[{c}_t\right]={\int}_0^t\left(j\left(t-s\right)+a\left(t-s\right)\right)f(s) ds.$$

Here, *f*(*s*) is the probability density function of the incubation period with a median of 28 days and a standard deviation of 9 days [10]. During the computation, we discretized the abovementioned models by week; thus, the integral ∫*ds* becomes the discrete sum ∑. Assuming that the observed incidence data followed a Poisson distribution, the likelihood function is

6 $$L\left({t}_{\theta },{R}_1,{\varepsilon}_1,{\varepsilon}_2;{\boldsymbol{x}}_t\right)={\Pi}_t\frac{E{\left[{c}_t\right]}^{x_t}{\exp}\left(-E\left[{c}_t\right]\right)}{x_t!},$$

given the observed number of cases *x*_t_ in week *t*. Here, *t*_*θ*_ is the week in which the first human-to-human transmission took place, and we assume that there were only environmental transmissions prior to *t*_*θ*_.

Because parameters *ε*_1_ and *ε*_2_ can vary with the serial interval, which is assumed to be known in the present study, it is vital to examine how these parameters take on different values if the serial interval is changed. As a sensitivity analysis, we estimated these parameters by varying the ratio of the standard deviation to the mean (i.e., the coefficient of variation, CV) of the serial interval. We also considered the following alternative model as part of sensitivity analysis, accounting for potential dependence between environmental and human-to-human transmissions, i.e.,

7 $$j(t)=R(t){\int}_0^t\left(j\left(t-s\right)+a\left(t-s\right)\right)g(s) ds,$$

where *R*(*t*) stands for the effective reproduction number at calendar time *t* and then,

8 $$E\left[{c}_t\right]={\int}_0^tj\left(t-s\right)f(s) ds.$$

For the original model, the following model assumptions were made. First, we assumed that the generation time followed a log-normal distribution. Second, preventive campaigns were assumed to abruptly influence the infection dynamics. The third assumption was that the two interventions independently influenced the epidemic dynamics, meaning that the combined impact could be modeled as the product of *ε*_1_ and *ε*_2_. Fourth, the epidemic dynamics were assumed to be a simple additive function of environmental transmission and human-to-human transmission, and the former was assumed to be approximated by a periodic function. Finally, we assumed that all infectious persons were diagnosed and reported.

### Ethical considerations

This study analyzed data that are publicly available. The datasets used in our study were de-identified and fully anonymized in advance. The analysis of publicly available data (Supplementary Data 1) without identity information does not require ethical approval.

## Results

**Fig. 3:**
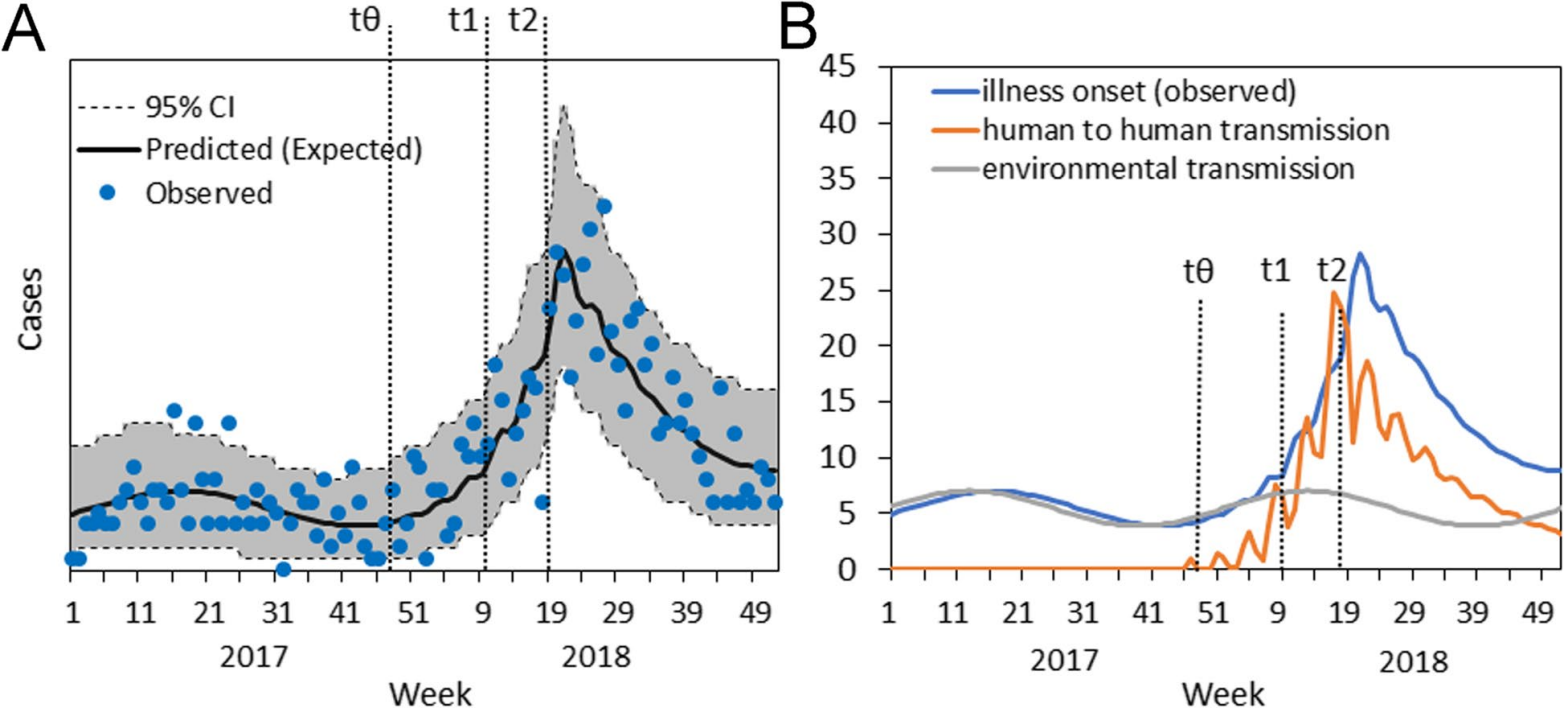
Decomposition of the epidemic curve. **A** Comparison of predicted and observed weekly incidence. The solid line is the expected weekly incidence of hepatitis A, and the dotted line represents the empirical data. The gray shaded area represents the 95% confidence interval calculated by the parametric bootstrap method. The three vertical lines represent the times at which (i) human-to-human transmission began to increase, (ii) the first countermeasure was implemented, and (iii) the second countermeasure was implemented. **B** Decomposed temporal distribution. The gray line shows the predicted incidence of environmental transmission, the orange line represents the predicted incidence of human-to-human transmission, and the blue line represents the mixture of these two mechanisms, obtained as a convolution of the mixed incidence and the incubation period

To quantify the seasonal model (Eq. 1), nine possible combinations were examined by varying *n*, the number of terms in the trigonometric function (0 to 2), and the length of learning data (2015–2017, 2016–2017, and 2017). Comparing Akaike information criterion (AIC) values, the minimum value was obtained when *n* = 1 and learning data from either 2016–2017 or 2017 were used. Across the examined learning data periods, the minimum AIC value was observed when *n* = 1; thus, we decided to adopt *n* = 1 in the model combining seasonal variation in environmental transmission and human-to-human transmission in the MSM population. Figure 3A compares the predictions using 2017 as learning data with the observed data, showing that most of the observed data were contained within the 95% confidence intervals (CIs) of the predictions. The human-to-human transmission epidemic was estimated to begin in the 35th week in 2017. Quantifying the transmission dynamics, we observed that the human-to-human transmission peaked around the time when the second countermeasure was implemented (Fig. 3B).

**Fig. 4:**
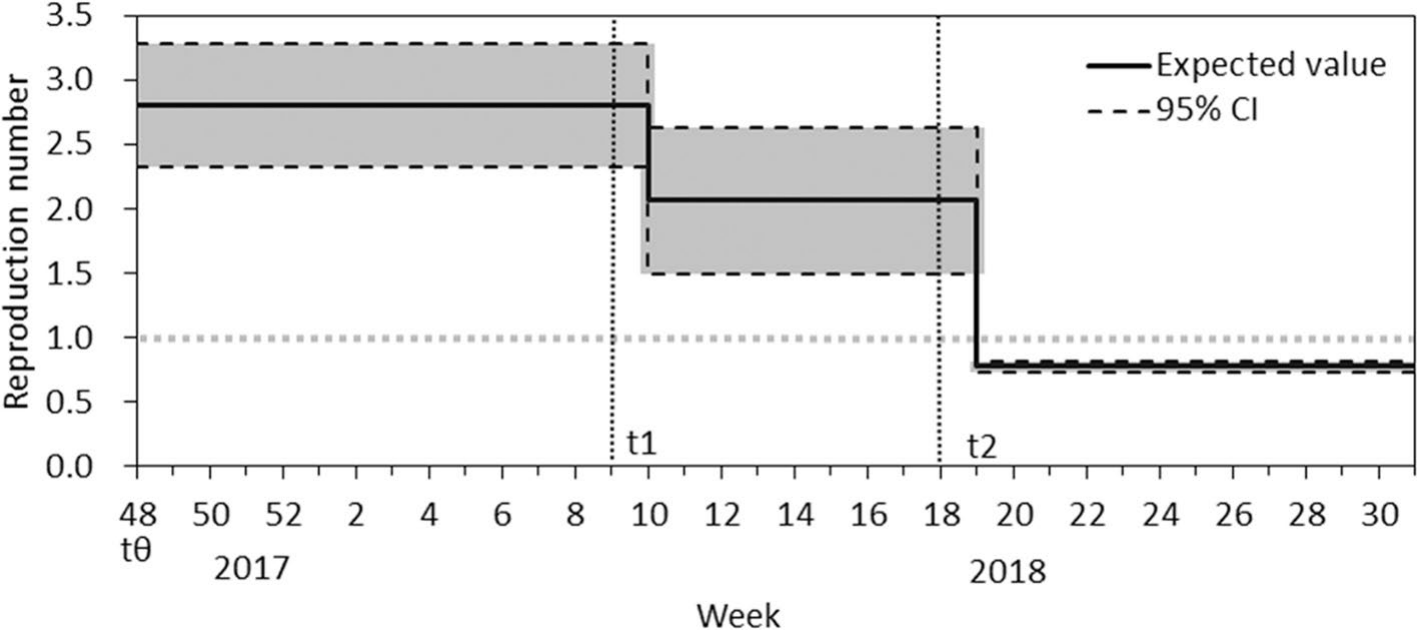
Estimated effective reproduction numbers. The solid line shows the reproduction number, and the gray shaded area shows the 95% confidence interval. The reproduction number decreased following the first and second countermeasures and, notably, fell below one following the second countermeasure

The estimated parameters along with their 95% CIs are summarized in Table 2. Overall, the presence of seasonality (i.e., *n* = 1 or 2) was favored, compared with the constant baseline (i.e., *n* = 0). The AICs using different learning data cannot be compared because of different amounts of empirical data across the time periods. While parameter values in Table 2 show those of public health interests, coefficients of trigonometric functions were also estimated, and the parameter estimates are available as Supplementary Data 2. In the following analyses, we present the results using the 2017 learning data because, although it was likely, we did not explicitly account for human-to-human transmission in the data from earlier years. The reproduction number before the interventions, *R*_1_, was estimated to range from 2.6 to 3.1, and *n* = 0 always yielded a greater *R*_1_ estimate compared with *n* = 1 or *n =* 2. The relative risk of transmission at the time of the first countermeasure, which involved the dissemination of pamphlets by non-governmental organizations associated with MSM, ranged from 0.59 to 0.87. Using the 2017 data with *n* = 1, *ɛ*_1_ was estimated at 0.72 (95% CI: 0.39–1.04). At the time of the second countermeasure, which used online web articles, the additional relative impact was estimated to range from 0.36 to 0.43. Using the 2017 data with *n* = 1, *ɛ*_2_ was estimated at 0.39 (95% CI: 0.27–0.52). Thus, the reproduction number was 0.72 × 0.39 = 0.28 times the baseline; accordingly, the reproduction number fell below one, and the incidence started to decline (Fig. 4). It should also be noted that the upper bound of the CI for the reproduction number fell below one following the second countermeasure.

**Table 2:** Estimated parameters and its 95% CI, time of outbreak, number of parameters, and AICa for each model assumption

Model assumption	*R*_1_^b^	*ε*_1_^c^	*ε*_2_^d^	Number of parameters	AIC
Constant, 17	3.05 (2.63,3.47)	0.60 (0.38,0.82)	0.41 (0.30,0.52)	5	566.0
Sine curve, 17	2.80 (2.32,3.29)	0.72 (0.39,1.04)	0.39 (0.27,0.52)	7	554.6
Second-order trigonometric, 17	2.80 (2.32,3.29)	0.72 (0.39,1.04)	0.39 (0.27,0.52)	9	558.6
Constant, 16-17	3.07 (2.66,3.49)	0.59 (0.37,0.80)	0.42 (0.30,0.53)	5	832.8
Sine curve, 16-17	2.74 (2.24,3.24)	0.76 (0.40,1.12)	0.38 (0.26,0.51)	7	797.8
Second-order trigonometric, 16-17	2.60 (2.07,3.13)	0.87 (0.42,1.33)	0.35 (0.23,0.47)	9	798.3
Constant, 15-17	3.13 (2.71.3.53)	0.57 (0.37,0.77)	0.43 (0.32,0.54)	5	1079,1
Sine curve, 15-17	2.82 (2.35,3.30)	0.72 (0.40,1.03)	0.39 (0.27,0.51)	7	1040.4
Second-order trigonometric, 15-17	2.68 (2.18,3.19)	0.82 (0.42,1.27)	0.36 (0.24,0.47)	9	1038.0

In all models, the estimated starting time of the outbreak was week 35 in 2017. Estimated values of parameters for trigonometric function are available from Supplementary Data 2

^a^Akaike Information Criteria

^b^reproductin number

^c^coefficient of the first measure

^d^coefficient of the second measure

^e^estimated time of invention of the first case of Hepatitis A by MSM

Applying the alternative model with time-dependent effect of interventions to 2015-17 data, sine function model was best-fit, and baseline *R*_1_ was estimated at 2.00. Parameters *ɛ*_1_ and *ɛ*_2_ were estimated to be 0.96 and 1.03, respectively. The AIC value of this model was 1183.6 which was greater than the original model with AIC=1040.4 (Table 2).

**Fig. 5:**
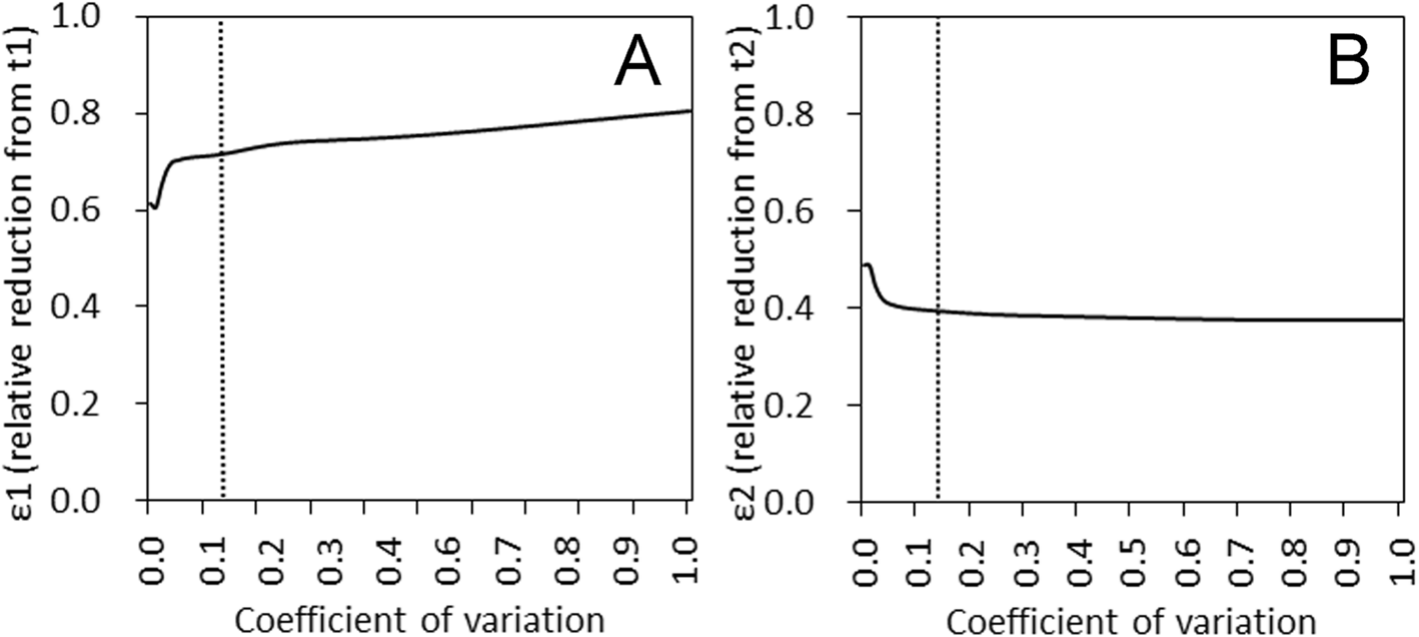
Sensitivity of the relative risk of transmission to serial interval variations. We fixed the median of the serial interval at 27.5 days [10]. In this figure, we varied the variance value, calculating the coefficient of variation to vary from zero to one. The vertical line shows the point that was used in the actual estimation

A sensitivity analysis was carried out. In particular, we examined how sensitive *ɛ*_1_ and *ɛ*_2_ were to variation in the CV of the serial interval (Fig. 5). Except for very small CV values, which would perhaps be unrealistic, the relative risk of transmission remained stable as CV varied. Thus, the estimated impact of the examined campaigns in terms of preventing hepatitis A transmission was shown to be robust to variation in the generation time, which remained uncertain in our model. Accounting for the possible dependence between environmental and human-to-human transmissions, the alternative model yielded subcritical value of *R*_1_, which is understandable for continued full involvement of environmentally infected individuals in the chains of transmission (but *R*_1_<1 was unlikely if the epidemic was induced mainly by contact in the population of MSM). For instance, using the data from 2015-17 and adopting a constant baseline, *R*_1_ was estimated at 0.83 (95% CI: 0.51, 1.14). AIC values of this alternative approach was always greater than those of original model.

## Discussion

The present study explored the epidemic of HAV that resulted in a surge of cases among adult men in 2018 in Japan [32]. During this epidemic, preventive campaigns were conducted to help avoid further transmission in the MSM population. Our study evaluated the effectiveness of these campaigns. Devising a mathematical model and estimating the parameters using surveillance data, we successfully captured the epidemic dynamics, quantifying the seasonal variation in environmental transmission. Prior to the countermeasures, the reproduction number ranged from 2.6 to 3.1; this value then started to decrease following the campaign-based interventions. After the first countermeasure, the reproduction number decreased, but the epidemic remained highly critical (i.e., *R*_*t*_ > 1). *R*_*t*_ fell well below one following the second countermeasure. Although the effective reproduction number, *R*_*t*_, changes because of both intrinsic and extrinsic factors, the present study suggests that the interventions that were implemented to raise situation awareness regarding the epidemic among the MSM population were influential. In contrast to the case in Japan, vaccination was required to control recent HAV epidemics in many other countries (e.g., the United States [30, 44] and Taiwan [35]).

In this study, we have objectively shown that *R*_*t*_ abruptly declined following the two examined campaign periods, although the possibility of intrinsic effects (e.g., saturation of infections because of clustering) cannot be excluded. Thus, our conclusion is conditional on the decline in *R*_*t*_ being attributed to extrinsic effects. However, we do not believe that the extent of transmission showed a clear clustering pattern, and the MSM population size is substantial; thus, it is likely that behavioral changes reduced risky contact. We examined the timing of two independent campaign periods. Of the two examined campaigns, the latter intervention, which involved publishing an online article, likely had a greater impact in terms of reducing the reproduction number. Given our study results, it would be valuable to survey the MSM population to investigate which types of input and messages were influential in promoting their behavioral changes.

What must be learned from this hepatitis A outbreak is that the MSM population in Japan is continuously exposed to the chance of HAV infection and that this risk is influenced by travelers visiting Japan. In this sense, although the examined campaigns were successful and no vaccination took place in this time period, it would be valuable to consider possible preventive vaccination against HAV. Acceptance of and demand for this vaccine should be surveyed in future studies.

As an important step forward in epidemiological modeling, we have modeled empirical data on HAV infection as a mixture of a renewal process and environmental transmission. This approach did not allow us to produce a closed-form likelihood, but we have numerically minimized the likelihood, modeling the illness onset data as a convolution of infection time-based incidence data and incubation period. Without understanding the likely timing of infection, it would not have been possible to explicitly evaluate the abrupt declines in *R*_*t*_. As for environmental transmission, seasonally fluctuating exposure was favored over assuming a constant hazard over time. This finding indicates the presence of seasonal variation, which may reflect exposure to seasonally varying risks (e.g., differences in eating and drinking). However, it must be noted that we assumed the absence of any outbreak among MSM during the control period; in this sense, there is room for improvement. Possible dependence between environmental and human-to-human transmissions was also examined, but AIC values of such models were greater than original models, and it was difficult to fully explain the plausibility of resulting parameters.

Several limitations must be acknowledged. First, we did not consider sexual contact or age, and future models should take these factors into account. Second, as an important direct indication of effectiveness, it would have been useful to monitor contact patterns directly. We used a step function to model *R*_*t*_, potentially reflecting contact patterns over time, but observing diminishing patterns of risky contact would provide more direct evidence for the effectiveness of the public health campaigns. Third, our model relied on surveillance notification data. There could be unreported cases, although underreporting of HAV infection is unlikely to be very frequent. Fourth, our model simulated a mixture of human-to-human and environmental transmission, and having genome and travel-history data would have allowed us to further disentangle the case data in a direct manner.

Despite these limitations, we believe the present study successfully shows that the timing of countermeasures against hepatitis A in the MSM population in Japan was consistent with abrupt declines in *R*_*t*_. This finding suggests that using online articles may have the potential to have widespread impact in terms of changing risky behaviors.

## Conclusions

The present study explored the HAV epidemic that resulted in a surge of cases among adult men in 2018 in Japan, as well as the preventive campaigns conducted to help avoid further transmission in the MSM population. The reproduction number prior to the countermeasures ranged from 2.6 to 3.1; this value began to decrease following the campaign-based interventions, falling well below one following the second countermeasure, which used an online article. Risky behaviors may have been changed by increasing situation awareness.

## Supplementary Information

**Additional file 1.**

**Additional file 2.**

## Data Availability

The original data used to construct the epidemic curve are available as Supplementary Data 1.

## Abbreviations

AIC: Akaike information criterion
CI: confidence interval
CV: Coefficient of variation
HAV: Hepatitis A virus
MSM: Men who have sex with men
